# Neodymium Laser Treatment for Overactive Bladder and Vulvodynia in Mayer-Rokitansky-Küster-Hauser Syndrome Patient: A Case Report

**DOI:** 10.7759/cureus.53068

**Published:** 2024-01-27

**Authors:** Nobuo Okui, Tadashi Ikegami, C.Tamer Erel

**Affiliations:** 1 Urology, Yokosuka Urogynecology and Urology Clinic, Yokosuka, JPN; 2 Diagnostic Imaging, Kanagawa Dental University, Yokosuka, JPN; 3 Obstetrics and Gynecology, Istanbul University, Cerrahpasa School of Medicine, Istanbul, TUR

**Keywords:** urinary urgency, neodymium yag laser therapy, vulvodynia, overactive bladder, mayer-rokitansky-küster-hauser syndrome

## Abstract

The Mayer-Rokitansky-Küster-Hauser (MRKH) syndrome is a condition that affects a small proportion of female individuals at birth, resulting in the absence or underdevelopment of reproductive organs. However, this case report introduces overactive bladder (OAB) and vulvodynia, conditions that have not been previously reported in MRKH patients. The 36-year-old patient began developing breast tissue around the age of 12 but never experienced menstruation. Simultaneously, she started experiencing discomfort in the genital region and frequent urination. These symptoms gradually worsened, making it difficult for her to continue her education, and initially, she was misdiagnosed with a developmental disorder. Typically, the general understanding of MRKH syndrome has focused on reproductive anomalies, but this case underscores its diversity. Diagnostic assessments, including ultrasound, MRI, and various tests, revealed that the patient's severe genital discomfort and urinary symptoms were improved through a specialized Neodymium YAG laser therapy named "PIANO mode," resulting in significant symptom relief and improved quality of life. This report emphasizes the importance of comprehensive and individualized approaches to managing MRKH syndrome. It aims to raise awareness that MRKH syndrome, while often associated with reproductive abnormalities, can also involve related symptoms like OAB and vulvodynia, which can significantly impact daily life.

## Introduction

Mayer-Rokitansky-Küster-Hauser (MRKH) syndrome is a rare condition related to the development of the female reproductive system, estimated to occur in approximately one out of every 5,000 live female births [1-3]. This syndrome is primarily attributed to the underdevelopment or absence of the paramesonephric ducts, which play a crucial role in the formation of the fallopian tubes, uterus, cervix, and upper two-thirds of the vagina. It typically manifests during adolescence and is defined by the absence of primary menstruation following the normal development of secondary sexual characteristics [4]. As a result, patients often report the absence of menstruation along with symptoms such as sexual difficulties, infertility, and periodic abdominal pain [3].

The pathogenesis of MRKH syndrome is complex and likely involves multiple factors contributing to tissue patterning and organ morphogenesis during human embryonic development [5]. This may include genetic factors, soluble morphogens, chemical cues, and mechanical forces, among others [6]. Consequently, the etiology of MRKH syndrome remains incompletely understood, and ongoing research seeks to unravel the underlying mechanisms.

The diagnosis of MRKH syndrome typically involves the identification of structural abnormalities in the uterus and vagina, although associated renal anomalies in the urinary system may also be present, necessitating renal assessments through techniques such as ultrasound and MRI [3].

In this case report, we present the case of a 36-year-old patient who has experienced symptoms of urgency in urination and vulvodynia since adolescence, in addition to the typical features of MRKH syndrome. This case underscores the diversity of symptoms associated with MRKH syndrome, beyond the focus on genital anomalies, emphasizing the need for holistic medical care.

## Case presentation

We would like to present the case of a 36-year-old female patient at our clinic. This patient began developing breast tissue at the age of 12 but has never experienced menstruation up to the present day. Concurrently, she began experiencing discomfort in the area leading to her genitals, along with frequent urination. These symptoms gradually worsened, leading to difficulties in continuing her education, and she was initially misdiagnosed with a developmental disorder. She attempted to enter the workforce but was hindered by severe discomfort in her genital area and urgency in urinating, preventing her from maintaining stable employment.

Approximately one year ago, she began experiencing abdominal pain in addition to discomfort in her genital area. Ultrasound examinations and internal examinations revealed the congenital absence of the uterus and vagina. Gynecologists prescribed various medications, including mirabegron at doses of 25 mg and 50 mg and fesoterodine fumarate at doses of 4 mg and 8 mg for her overactive bladder, but there was no improvement. An experimental approach involving female hormone replacement therapy was attempted, resulting in severe mastitis. Unfortunately, her genital pain was not addressed during this process. She was also considered to have a developmental disorder and was prescribed anxiolytic medication, which did not alleviate her symptoms. Her life became increasingly challenging.

She sought treatment at our clinic for her genital pain and urinary urgency, which significantly diminished her quality of life. Although she had an interest in romantic relationships with men, she was in a state where she had no mental capacity to consider vaginal reconstruction surgery. She expressed that she did not believe she had a developmental disorder and that if her genital pain and urinary urgency could be resolved, she could continue with employment.

She stands at a height of 159 cm and weighs 56 kg. The Attention Deficit Hyperactivity Disorder Rating Scale-IV was within the normal range, and there were no signs of hyperactivity or developmental disorders. Her general physical examination revealed no abnormalities, and her other vital signs remained stable. She maintains a well-balanced diet, has no history of smoking, and does not consume alcohol. She has no previous medical records and has never undergone surgery.

Breast examination indicated Tanner stage 5 development for both breasts, which is consistent with her age. Examination of her external genitalia showed normal labia majora, labia minora, appropriate pubic hair development, and a normal external urethral meatus.

**Figure 1:**
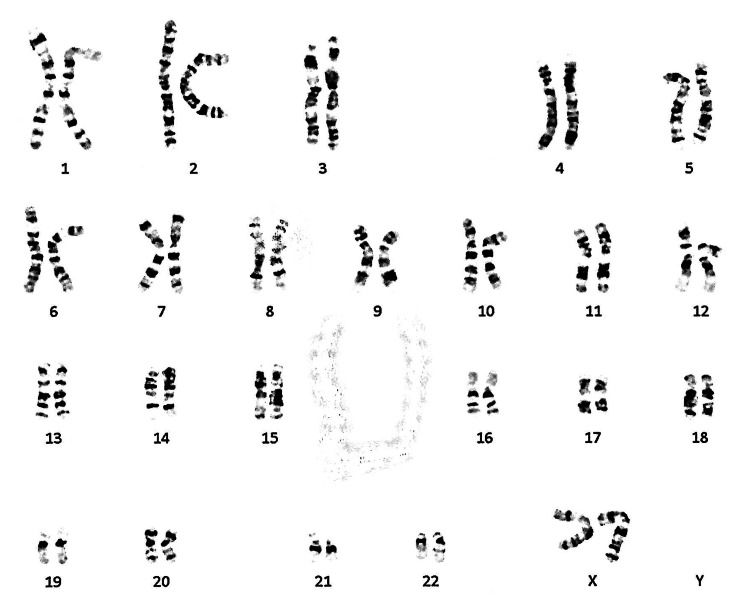
The peripheral blood karyotype

Blood tests revealed a total leukocyte count of 5390/μL, a platelet count of 24,000/μL, a hemoglobin level of 12.8 gm%, blood urea nitrogen of 14.6, creatinine at 0.62 (Table 1), and a peripheral blood karyotype of 46XX (Figure 1). Additional laboratory parameters can be found in Table 1. Hormone levels, including estradiol, progesterone, luteinizing hormone, follicle-stimulating hormone, prolactin, and testosterone, were all within the normal range (Table 1).

**Table 1:** Laboratory parameters of the patient WBC: white blood cells; HB: hemoglobin; HCT: hematocrit; PT: prothrombin time; BS: blood sugar; BUN: blood urea nitrogen; LH: luteinizing hormone; FSH: follicle-stimulating hormone; HIVAb: human immunodeficiency virus antibody; HBsAg: hepatitis B virus surface antigen; HCVAb: hepatitis C virus antibody

Test	Result	Unit	Reference range
WBC	5,390	/μL	4,000–11,000
HB	12.7	g/dL	12.5–15.0
HCT	40.1	%	37.5–45
Platelets	24,000	/μL	37,900–140,000
Neutrophils	68	%	45–75
Lymphocytes	27	%	25–45
Monocytes	0	%	2–10
Eosinophils	6	%	1–6
Basophils	0	%	0–1
PT	13	seconds	10–12
Blood sugar	5.2	mmol/L	3.8–7.8
BUN	14.5	mg/dL	8–20
Creatinine	0.62	mg/dL	0.46–0.82
Sodium	140.1	mEq/L	135–146
Potassium	4.0	mEq/L	3.5–5.2
Estradiol	366	pg/ml	19-226 (follicular phase)
			49-487 (ovulation period)
			78-252 (luteal phase)
			<39 (postmenopausal)
Progesterone	8.8	ng/ml	<0.4 (follicular phase)
			<3.7 (ovulation period)
			8-21 (luteal phase)
LH	11.2	mIU/ml	1.8-10.2 (follicular phase)
			2.2-88.3 (ovulation period)
			1.1-14.2 (luteal phase)
			5.7-64.3 (postmenopausal)
FSH	6.4	mIU/ml	3.0-14.7 (follicular phase)
			3.2-16.6 (ovulation period)
			1.5-8.5 (luteal phase)
			<157.8 (postmenopausal)
Prolactin	22.2	pg/ml	6.1-30.5
Testosterone	33	ng/dl	10.8-56.9
HIVAb	Non-reactive		
HBsAg	Non-reactive		
HCVAb	Non-reactive		

Ultrasound examinations confirmed the absence of the uterus, cervix, and vagina, as well as the normalcy of both kidneys.

The patient experienced constant discomfort in the genital area, along with a persistent urge to urinate. According to a three-day urinary diary, she was urinating more than 30 times a day. Activities such as sitting on a chair and performing desk work, which stimulated the genitalia and urethra, increased her frequency of urination. Swab tests of the genital area, especially around the urethra, produced pain due to stimulation.

In uroflowmetry studies, the maximum urine flow rate (Qmax) was 45 mL/s, the voided volume (VV) was 132 mL, and no residual urine was detected. During the cystometric study, the patient reported urethral pain upon catheter insertion, which persisted throughout the examination. The initial bladder pressure and urethral pressure at the start of the infusion were 33.8 and 28 cm H_2_O, respectively. Although the urethra was painful, there were no strong sphincter contractions or urethral obstructions. She reported a first desire (FD), normal desire (ND), and maximum capacity (MCC) of 62 mL, 112 mL, and 202 mL, respectively. Despite presenting symptoms of an overactive bladder, no detrusor overactivity was observed during the cystometric study. These tests were conducted using the Goby Family of Wireless Urodynamics Systems (EDAP TMS SA, Rhône, France).

The cystoscopy revealed no lesions in the bladder. The ureteral orifices appeared normal, and there were no ureteral diverticula present. The labia were well-developed, and the patient reported no issues with masturbation. The vaginal opening did not have a hymen, and the corresponding area was identified based on mucosal changes, with the vagina having a depth of approximately 1 cm.

After counseling and obtaining approval from the Institutional Review Board, informed consent was obtained from the patient in writing, and the patient chose to undergo laser therapy (L1 and L2) at three-month intervals. Follow-up was conducted three months after the final laser therapy session. Neodymium Yttrium Aluminum Garnet (Nd:YAG) treatment was performed at an outpatient clinic. Prior to the procedure, the patient's genitalia were cleaned with an antiseptic solution and dried with a cotton swab. A 1064 nm Nd:YAG (PIANO mode, Fotona SP Dynamis, Fotona d.o.o., Ljubljana, Slovenia) was used with a spot size of 9 mm, PIANO pulse mode (five seconds), and a fluence of 90 J/cm^2^. Six passes were performed in brushing mode to treat the entire external genitalia [6]. The patient was advised to refrain from masturbation for one week following the treatment. The treatment was performed without anesthesia as it is practically painless, and there were no adverse effects reported from this treatment.

The patient's symptoms improved with each follow-up. After two sessions of Nd:YAG therapy (L1, L2), surveys were conducted at one month (T1) and three months (T3). The urination interval at T1 was longer than at T0 and remained the same at T3. The urinary frequency, which was 30 times/day at T0, decreased to 10 times/day at T1 and eight times/day at T3, with a similar daily fluid intake.

**Figure 2:**
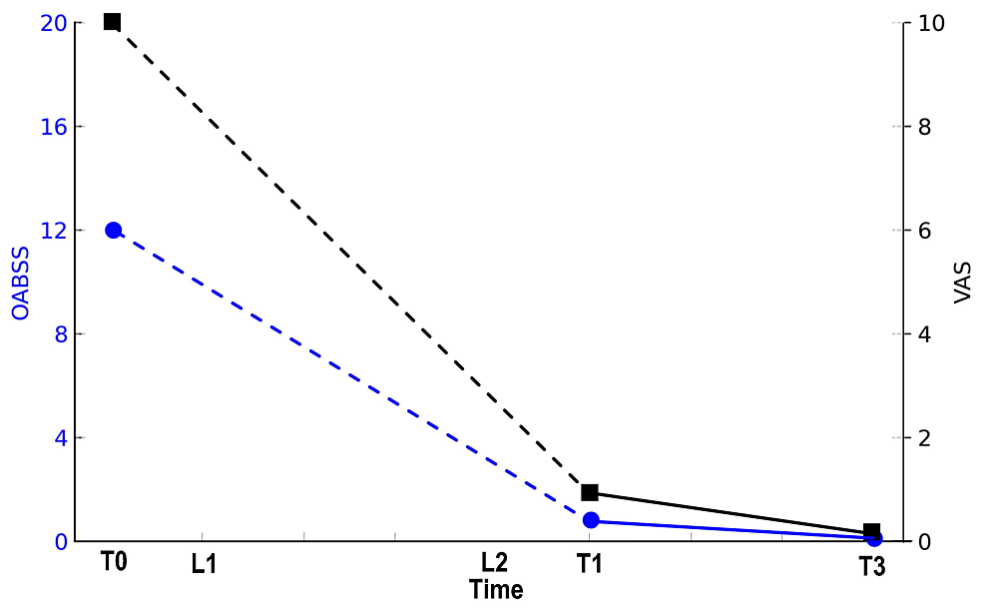
Changes in OABSS and VAS OABSS: Overactive Bladder Symptom Score; VAS: Visual Analog Scale; T0: before treatment; T1: one month after treatment; T3: three months after treatment; L1: the first laser therapy; L2: the second laser therapy Blue Closed Circle: OABSS and Black Closed Square: VAS The left Y-axis represents the OABSS, while the right Y-axis corresponds to the VAS. In L1 and L2, OABSS and VAS were not measured, so the line between T0 and T1 is dotted. The X-axis represents the passage of time, indicating the time points T0, L1, L2, T1, and T3.

The Overactive Bladder Symptom Score (OABSS), an indicator of overactive bladder (OAB), decreased from 12 at T0 to 1 at T1 and 0 at T3. The patient experienced significantly reduced pain during urination, with the Visual Analog Scale (VAS) score improving from 10 at T0 to 1 at T1 and 0 at T3 (Figure 2).

In the uroflowmetry study, there was no change in Qmax, but VV improved to 270 mL. During the cystometric study, the urethral pain that occurred after treatment disappeared. FD increased to 120 mL, ND to 220 mL, and MCC to 420 mL, with a particularly noticeable reduction in pain at MCC.

The patient underwent a comparison of pelvic organs before and after treatment using a 1.5T MRI (Signa Creator, GE Healthcare, Chicago, USA).

**Figure 3:**
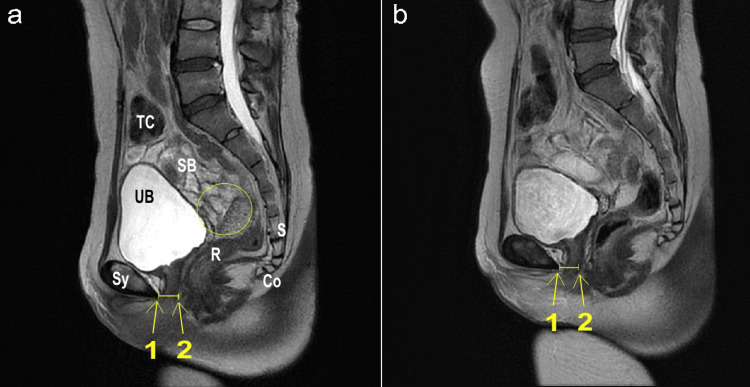
Changes in urethral thickness on T2-weighted sagittal view a: before treatment; b: after treatment Sy: symphysis pubis; UB: bladder; SB: small intestine; R: rectum; S: sacrum; Co: coccyx; TC: transverse colon Yellow open circle: normal location of the uterus The yellow line represents the distance between the posterior end of the pubic bone (1) and the posterior end of the urethra (2).

Figure 3 is an MRI in T2-weighted sagittal view (TR4500 ms, flip angle 140 degrees, TE 100 ms, slice thickness 3 mm, interval 3.5 mm, FOV 32x36 cm, Matrix 320x320). There is no signal of the uterus observed in the Douglas pouch. Compared to before treatment (Figure 3a), after treatment (Figure 3b), the distance between the posterior end of the pubic bone and the posterior end of the urethra has increased from 15mm to 17mm. Additionally, there is a noticeable change in the fat layer on the posterior side of the urethra.

**Figure 4:**
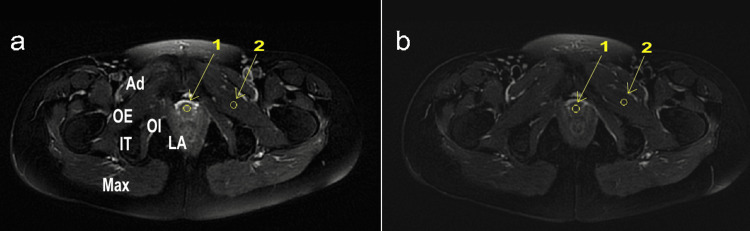
Changes in signal intensity ratio between the urethra and external sphincter on T2-weighted imaging a: before treatment; b: after treatment Ad: adductor muscles; OE: obturator externus muscle; IT: ischial tuberosity; OI: obturator internus muscle; LA: levator ani muscle; Max: gluteus maximus muscle The yellow circles represent the measurement of MRI values at the same location of the urethra (1) and OE (2).

Figure 4 is an MRI in the axial view with fat suppression in T2-weighted imaging (TR 4500 ms, flip angle 140 degrees, TE 118 ms, slice thickness 5 mm, interval 6 mm, FOV 42x47 cm, Matrix 384x384). When examining the ratio of MRI signals at the same location of the pelvic floor muscles and the urethra before treatment (Figure 4a), the ratio is 235/65=3.62. In contrast, after treatment (Figure 4b), the ratio is 114/55=2.07. This change indicates a decrease in the signal intensity ratio between the urethra and the pelvic floor muscles.

**Figure 5:**
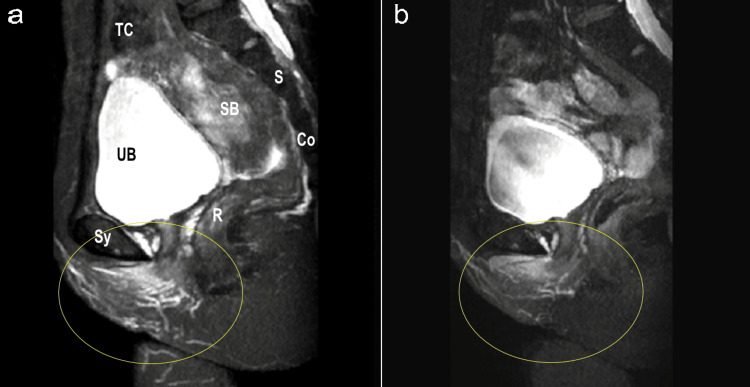
Changes in perineal congestion on heavy T2-weighted imaging a: before treatment; b: after treatment Sy: symphysis pubis; UB: bladder; SB: small intestine; R: rectum; S: sacrum; Co: coccyx; TC: transverse colon Yellow open circle: the venous congestion of blood

Figure 5 is an MRI, reconstructed to sagittal 8-mm-thickness images after MIP treatment using 3D data acquisition of T2-weighted coronal images with fat suppression (TR 3000 ms, TE 100 ms, FOV 42 x 47 cm, Matrix 384x256). Compared to before treatment (Figure 5a), after treatment (Figure 5b), there is a decrease in the signal of the veins in the urethra, anterior and posterior to it, and in the perineum.

After treatment, the patient's genital discomfort and overactive bladder symptoms disappeared, allowing her to resume her daily life without hindrance. The patient was able to find employment and has aspirations to resume her studies in the future.

## Discussion

MRKH syndrome was described over 130 years ago by Mayer, Rokitansky, Küster, and Hauser, initially focusing on uterovaginal agenesis without extragenital malformations (Type I MRKH). A deeper understanding developed through the 19th-century research of Mayer and Rokitansky, the first report of a living patient by Küster in 1910, and Hauser's comprehensive definition in 1961. In 1977, Schmid-Tannwald and Hauser identified cases with renal malformations (atypical MRKH or Type II), and Duncan et al. described a severe phenotype with renal and skeletal problems (MURCS association) [7,8]. Types I and II MRKH syndrome represent 56-72% and 28-44% of cases, respectively, with Type II including any extragenital abnormalities [9]. In this case, it was identified as uterovaginal agenesis without extragenital malformations, which is considered to be the most common type.

Patients with MRKH syndrome typically discover their condition during adolescence due to primary amenorrhea [10]. They often experience normal puberty and secondary sex characteristics. Common symptoms include dyspareunia and abdominal pain, with the average age at diagnosis being about 17.5 years [11]. Chromosome analysis is performed to confirm a normal female karyotype, and hormone levels are usually normal, though recent studies suggest the possibility of hyperandrogenemia in some patients [12]. In this case, normal chromosomes and hormone levels were observed.

MRKH syndrome in patients typically involves two aplastic uterine buds or complete Müllerian duct absence [3]. About 48-95% retain uterine remnants, potentially causing cyclic abdominal pain [13]. Surgery may be needed. Ovarian anomalies are rare and often more cranial. MRKH is categorized into Type I (no extragenital issues) and Type II (with abnormalities) [3]. Renal malformations are common, followed by skeletal issues and, less frequently, cardiac and hearing problems. Severe cases may have multiple congenital anomalies [14]. In this case, the presence of aplastic uterine buds suggests that the "itchy feeling" in the genital area and overactive bladder felt since the age of 12 is likely unrelated to Müllerian duct anomalies.

The diagnosis of MRKH syndrome can have a significant psychological impact, especially during adolescence [3,15-17]. It may lead to issues related to identity, sexuality, and infertility. Studies indicate that patients with MRKH syndrome commonly experience higher levels of psychological stress, including neuroticism and coping challenges, compared to women without the condition [3,15-17]. In this particular case, unlike typical reports, psychological issues stemmed from difficulties in schooling and employment due to the "itchy feeling" and overactive bladder, leading to a misdiagnosis of developmental disorders. This highlights the importance of recognizing the co-occurrence of vulvodynia and overactive bladder in MRKH syndrome.

Typically, in MRKH syndrome, creating a functional neovagina is a primary treatment goal, with various surgical and non-surgical methods developed over time [3,18]. However, in this case, the initial goal was not to create a new vagina but to treat vulvodynia and overactive bladder to enable employment. Furthermore, since the cause of the overactive bladder was vulvodynia, the bladder was not the target, as indicated by the MRI. Treatments targeting the bladder for overactive bladder were unnecessary and ineffective. In this case, Nd:YAG laser treatment proved effective. This is the newest treatment for vulvodynia, promoting tissue regeneration [19,20].

The Nd:YAG laser used in this case reacts with hemoglobin. Particularly, the PIANO mode used generates heat from the reaction between the laser energy and hemoglobin, causing vascular dilation. Previous studies have shown improvement in pain for vulvodynia, but reports are limited [19,20]. In this case, significant changes were observed in urethral thickness, signal intensity ratio, and vascular signals in the perineal area after Nd:YAG laser treatment. These changes suggest the influence of the Nd:YAG laser. From this evidence, it is believed that the thermal effects of the laser affect the sensitivity of nerve endings, alter the pain threshold, accelerate cell activation and the repair process, and contribute to improved tissue oxygen supply and nutritional status through angiogenesis.

The insights gained from this case indicate the need to explore new treatments for MRKH syndrome patients in addition to traditional methods. Furthermore, the treatment suggests a potentially effective approach to managing chronic pain and urinary urgency in MRKH syndrome, promising advancements in future research in this field. Future studies should verify the durability of the Nd:YAG laser treatment's effects and generalize its efficacy across a broader MRKH patient population. Detailed research into the mechanisms of treatment efficacy is also needed. Such studies are expected to contribute to the development of new treatment strategies for improving the treatment methods and quality of life for MRKH syndrome patients.

## Conclusions

In this case study, we explored the impact of associated symptoms such as vulvodynia and overactive bladder on a patient with MRKH syndrome, emphasizing how these can significantly affect daily life and should not be overlooked. Our findings show that Nd:YAG laser therapy was effective in addressing the patient's severe genital discomfort and urinary urgency, markedly improving her quality of life. This innovative approach challenges conventional treatment methods and suggests new possibilities for managing chronic pain and urinary symptoms in MRKH syndrome patients. The changes observed in VAS, OABSS, and MRI in our study highlight novel aspects of treatment for comorbidities in MRKH syndrome. Additionally, this case report underscores the importance of comprehensive and patient-centered care in MRKH syndrome, addressing symptoms that may otherwise be misunderstood as developmental disorders.
